# Severe *Staphylococcus lugdunensis* keratitis

**DOI:** 10.1007/s15010-014-0669-2

**Published:** 2014-08-01

**Authors:** N. Inada, N. Harada, M. Nakashima, J. Shoji

**Affiliations:** Division of Ophthalmology, Department of Visual Sciences, Nihon University School of Medicine, 30-1 Oyaguchi-Kamichou, Itabashi-ku, Tokyo, 173-8610 Japan

**Keywords:** *Staphylococcus lugdunensis*, Suppurative keratitis, Empiric therapy, Antibiotics

## Abstract

We report a severe case of *Staphylococcus lugdunensis* (*S. lugdunensis*) keratitis presenting as suppurative keratitis in a 77-year-old woman. The patient’s chief complaint was eye pain and decreased visual acuity in her right eye. Suppurative keratitis with a severe corneal abscess was diagnosed by a slit-lamp ophthalmic examination. The causative organism was identified as *S. lugdunensis* by bacterial culture, using a corneal abrasion specimen. She was treated with an intravenous drip infusion of ceftazidime and instillation of gentamicin sulfate ophthalmic solution (six times daily) and ofloxacin ophthalmic ointment (once daily before bedtime) as empiric therapy. Her hospital course was complicated by a corneal perforation of her right eye. The antibiotic susceptibility for *S. lugdunensis* was sensitive, but with a slightly high MIC for antibiotics used in empiric therapy. The therapeutic drug was changed to levofloxacin ophthalmic solution. The corneal abscess left a scar after healing. Representative causative organisms of suppurative keratitis include *Pseudomonas aeruginosa* and *Streptococcus pneumoniae*, but care must be taken in cases involving rare causative organisms. Empiric therapy is necessary for rapidly progressing suppurative keratitis, but a detailed examination of the causative organism is important for therapeutic planning before empiric therapy.

## Introduction

We report a severe case of bacterial keratitis in which *Staphylococcus lugdunensis* was identified as the causative pathogen. Bacteria in the genus *Staphylococcus* are classified as coagulase-positive staphylococci or coagulase-negative staphylococci (CNS). Coagulase-positive staphylococci, which include *S. aureus*, cause infections of the anterior segment of the eye, such as blepharitis, acute conjunctivitis, and corneal ulcers [1]. Meanwhile, in addition to residing in the conjunctival sac as indigenous bacteria, CNS may be implicated in infectious keratitis [1]. *S. lugdunensis* is a coagulase-negative staphylococcus that causes a rare but destructive form of infective endocarditis and skin and soft tissue infections [2, 3]. However, while the virulence of *S. lugdunensis* has many similarities to that of *S. aureus*, its drug-sensitivity and disease severity are different from those of other CNS pathogens. However, thus far, *S. lugdunensis* has been rarely isolated and identified from suppurative keratitis lesions, and details regarding the clinical course of infection have not been reported. In this paper, we report the clinical course of *S. lugdunensis*-induced suppurative keratitis.

## Case report

**Fig. 1:**
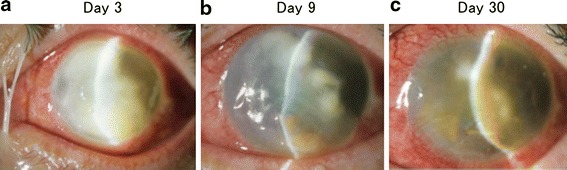
Slit-lamp microscope photographs demonstrate the clinical course of *S. lugdunensis* keratitis. **a** Day 3: abscess formation is present on the entire corneal surface, and the anterior chamber could not be transilluminated. **b** Day 9: an anterior chamber formed again after a corneal perforation. The corneal abscess shows a tendency toward remission. **c** Day 30: the epithelial deficiency has disappeared, and a reduction of the abscess and corneal neovascularization are present

The patient was a 77-year-old Japanese woman who was referred to our hospital because of severe discharge, pain, and decreased visual acuity in her right eye. Her symptoms developed 3 days after farming, and she consulted our institution. The findings at the time of the initial visit (day 3) were as follows. She did not have any specific ophthalmological or systemic anamnesis other than decreased visual acuity resulting from excessive myopia. The best-corrected visual acuity was light perception. Slit-lamp microscopy findings showed suppurative keratitis with corneal abscess formation over the entire surface of the cornea, and the anterior chamber could not be transilluminated (Fig. 1a). We performed scrapings of the corneal abscesses using a spatula to obtain smear and culture specimens. Then, the corneal smear was Gram stained and the corneal scrapings were placed directly onto chocolate agar. The bacterial culture was placed in a carbon dioxide incubator at 35 °C for 5 days. The identification of *S. lugdunensis* was performed using N-ID Test SP-18 “Nissui” (Nissui Pharmaceutical, Tokyo, Japan) [4]. This test kit can identify any strain of *Staphylococcus* Rosenbach through a combination of 18 different biochemical properties. The tests included acid production from fructose, mannose, maltose, lactose, trehalose, mannitol, raffinose, sucrose, *N*-acetylglucosamine, turanose, ribose, and arabinose; decarboxylation of arginine; production of urease, β-glucuronidase, acetoin, and alkaline phosphatase; and reduction of nitrate. Moreover, antibiotic susceptibility tests were performed using RAISUS ANY (Nissui Pharmaceutical) [5].

Treatment was performed using an empiric therapy involving an intravenous drip infusion of ceftazidime (Modacin^®^ for injection, GlaxoSmithKline, Tokyo, Japan) at a dose of 1 g per infusion administered twice daily, as well as gentamicin sulfate ophthalmic solution (Riftamycin^®^ ophthalmic solution 0.3 %, Wakamoto, Tokyo, Japan) administered six times daily and ofloxacin ophthalmic ointment (Tarivid^®^ ophthalmic ointment 0.3 %, Santen, Osaka, Japan) administered once daily before bedtime. The corneal abscess showed initial improvement with therapy; however, on disease day 9, thinning of the central part of the cornea led to perforation (Fig. 1b). Later, the perforated site closed spontaneously. After a clinical course of approximately 2 weeks, remission of the abscess occurred, as well as healing with scar formation (Fig. 1c). A culture of the corneal scrapings, which showed no organisms on a Gram stain, yielded *S. lugdunensis*, and drug susceptibility tests showed no drug resistance (Table 1). In a chocolate agar culture to which the corneal scraping was directly applied, fungi and bacteria were not detected, except for *S. lugdunensis*. The therapeutic drug was changed to levofloxacin ophthalmic solution (Cravit^®^ ophthalmic solution 0.5 %, Santen, Osaka, Japan) according to the results of the bacterial culture and antibiotic susceptibility tests (Table 1). The corneal abscess left a scar after healing.

**Table 1:** Antibiotic susceptibility of *Staphylococcus lugdunensis*

ABPC	ABPC/SBT	OX	CEZ	IPM/CS	ABK	GM
≤0.25	≤8	≤0.25	≤8	≤4	≤0.5	≤4

CAM	CLMD	MINO	VCM	TEIC	ST	LVFX
≤0.5	≤0.25	≤2	≤1	≤2	≤20	≤1

Values are expressed in MIC (µg/mL)

*ABPC* ampicillin, *SBT* sulbactam, *OX* oxacillin, *CEZ* cefazolin, *IPM/CS* imipenem/cilastatin, *ABK* arbekacin, *GM* gentamicin, *CAM* clarithromycin, *CLMD* clindamycin, *MINO* minocycline, *VCM* vancomycin, *TEIC* teicoplanin, *ST* sulfamethoxazole/trimethoprim, *LVFX* levofloxacin

## Discussion

The present case involved suppurative keratitis caused by *S. lugdunensis* in an elderly patient, and the characteristic findings of our case are as follows: (1) the contributing factors are unknown, (2) the condition can lead to severe suppurative keratitis, (3) the clinical course is rapid, and (4) the infectious corneal ulcer showed susceptibility to treatment with antibiotics. The representative causative bacteria of suppurative keratitis are *Streptococcus pneumoniae* and *Pseudomonas aeruginosa* [6, 7], and suppurative keratitis progresses rapidly. Therefore, in the treatment of suppurative keratitis, it is important that empiric therapy be initiated without waiting for the results of a bacterial culture. The antibacterial spectrum of antibiotics is taken into consideration when conducting empiric therapy; when the causative bacterium is presumed to be a gram-positive coccus such as *Streptococcus pneumoniae*, a treatment combining cephem and fluoroquinolone antibacterial agents in an ophthalmic solution is selected, and when the causative bacterium is presumed to be a gram-negative bacillus such as *Pseudomonas aeruginosa*, a treatment combining aminoglycoside and fluoroquinolone antibacterial agents in an ophthalmic solution is selected. In the present case, the clinical examination revealed severe suppurative keratitis; therefore, empiric therapy was conducted presuming that the causative bacterium was *Pseudomonas aeruginosa*. However, the efficacy of the gentamicin and ofloxacin treatment used in this case will need to be verified. *S. lugdunensis* was previously isolated from a patient with suppurative keratitis [5], suggesting it should be recognized as a causative organism of suppurative keratitis. The results of drug-sensitivity tests conducted on the clinical isolates in the present case showed no drug resistance, but low sensitivity to gentamicin, which was used in the empiric therapy. In addition, β-lactamase-producing strains of *S. lugdunensis* have been isolated from abscesses and surgical wounds [3], suggesting that certain strains may exhibit drug resistance similar to that of methicillin-resistant *S. aureus*. Therefore, before conducting an empiric therapy, scrapings of the lesions are recommended to identify the causative organism through smear testing and bacterial isolation and culture, and the antibiotic therapy may need to be revised in accordance with the test results. The small amount of corneal scraping specimens that can be collected limits the choice of microbiological methods that can be exploited in diagnosis. Accordingly, we performed gram staining using a smear and a chocolate agar culture in this examination. Chocolate agar is a bacterial isolation medium, but may also be able to isolate fungi such as *Candida* and *Fusarium* in about 5 days of culture (data not shown). Therefore, at our institution, we directly apply a corneal scraping specimen to chocolate agar as a screening test for infectious keratitis when a small amount of specimen is obtained.

*Staphylococcus lugdunensis*-induced keratitis manifests as acute suppurative keratitis, and selecting antimicrobial agents on the basis of drug-sensitivity tests is essential for treatment.
